# Developing a national musculoskeletal core capabilities framework for first point of contact practitioners

**DOI:** 10.1093/rap/rkz036

**Published:** 2019-09-20

**Authors:** Kenneth Chance-Larsen, Michael R Backhouse, Richard Collier, Colin Wright, Sally Gosling, Beverley Harden, Sarah Marsh, Peter Kay, Hilary Wyles, Jo Erwin, Anthony Woolf

**Affiliations:** 1 School of Health Sciences, University of Central Lancashire, Preston; 2 Leeds Institute of Rheumatic and Musculoskeletal Medicine, University of Leeds, Leeds; 3 York Trials Unit, Department of Health Sciences, University of York, York; 4 Academy for Advanced and Consultant Practice, Health Education England, London; 5 Core Skills Frameworks, Skills for Health, Bristol; 6 Practice and Development, Chartered Society of Physiotherapy, London; 7 Clinical Policy Unit, NHS England, London; 8 Bone & Joint Research Group, Royal Cornwall Hospitals NHS Trust, Truro; 9 Arthritis and Musculoskeletal Alliance (ARMA), London, UK

**Keywords:** musculoskeletal, core capabilities, first point of contact

## Abstract

**Objective:**

We aimed to support service transformation by developing a core capabilities framework for first contact practitioners working with people who have musculoskeletal conditions.

**Methods:**

We conducted a modified three-round Delphi study with a multi-professional panel of 41 experts nominated through 18 national professional and patient organizations. Qualitative data from an open-ended question in round one were analysed using a thematic approach and combined with existing literature to shape a draft framework. Participants rated their agreement with each of the proposed 142 outcomes within 14 capabilities on a 10-point Likert scale in round two. The final round combined round two results with a wider online survey.

**Results:**

Rounds two and three of the Delphi survey were completed by 37 and 27 participants, respectively. Ninety practitioners responded to the wider online survey. The final framework contains 105 outcomes within 14 capabilities, separated into four domains (person-centred approaches; assessment, investigation and diagnosis; condition management, intervention and prevention; and service and professional development). The median agreement for all 105 outcomes was at least nine on the 10-point Likert scale in the final round.

**Conclusion:**

The framework outlines the core capabilities required for practitioners working as the first point of contact for people with musculoskeletal conditions. It provides a standard structure and language across professions, with greater consistency and portability of musculoskeletal core capabilities. Agreement on each of the 105 outcomes was universally high amongst the expert panel, and the framework is now being disseminated by Health Education England, NHS England and Skills for Health.

**Key messages**

- The framework describes the capabilities required for practitioners working in first point of contact roles.
- The framework can be used by commissioners, service, education and training providers and practitioners.
- Musculoskeletal practitioners can use the framework to map skills and learning needs to role requirements.

## Introduction

It is estimated that 17.8 million people in the UK live with a musculoskeletal (MSK) condition and that these conditions remain the leading cause of years lived with disability and the third largest cause of disability-adjusted life years in the UK today [1]. Musculoskeletal conditions are the second largest cause of sickness absence in the UK; they are responsible for the loss of >30 million working days per annum [2], and there is a significant impact on employment rates of people with an MSK condition [3]. Musculoskeletal conditions cost the National Health Service (NHS) £4.76 billion in 2013–14, the third largest area of NHS spending [4], and place a significant burden on general practitioners (GPs), accounting for 30% of consultations in England [5].

The combination of an ageing population and rising levels of obesity means that the burden of MSK conditions is likely to increase over the coming years [6]. This will place further pressure on already stretched GP services and requires new approaches and health service transformation to meet the changing needs of the population. One recent innovation has been the emergence of first contact practitioners (FCPs), which aims to place skilled MSK clinicians, typically from non-medical backgrounds, earlier in the patient pathway, with the aims of improving patient outcomes and reducing GP workload.

Musculoskeletal FCP roles have developed primarily in GP practices, and initial reports suggest a positive impact for both patients/service users and the health-care provider. This includes better clinical outcomes, less prescribing, more appropriate onwards referrals, better conversion rates for surgery and high patient satisfaction scores [7, 8]. Additionally, the MSK FCP roles have been shown to reduce MSK-related GP practice cost and free up GP capacity [7–9]. It is worth noting, however, that the overall GP workload does not seem to be reducing. The number of monthly GP appointments in England increased by >82 000 to 12 592 229 in May 2019 compared with a year earlier, despite a reduction in the number of open active practices [10]. Increasingly, services are being reconfigured to place non-medical MSK FCPs earlier in the patient pathway. The NHS Long term Plan [11] reports that 98% of the Sustainability and Transformation Partnerships in England have confirmed pilot sites for MSK FCPs, and 55% of these are currently underway. The new NHS England GP contract also outlines 70% funding for an estimated 20 000 additional staff by 2023/24, including first contact physiotherapists [12].

The benefits of having one capability framework in this domain presents several advantages, including consistency across professions and portability between roles, and negates the need for the busy MSK practitioner to have to relate to numerous frameworks in their day-to-day practice. A common MSK framework can provide clarity on the expected standards of service delivery, and details of the knowledge, skills and behaviours that health-care practitioners need to develop and demonstrate. The drivers for the development of the framework include policy [13], the national work programme delivered by the Arthritis and Musculoskeletal Alliance (ARMA) and its member organizations [14] working in partnership with NHS England, with the National Clinical Director for MSK Services and the Elective Care Transformation Programme [15].

Existing frameworks typically use the term competence to describe the required skills, knowledge and behaviours by health-care practitioners. We decided that capabilities better describe what these practitioners should be able to do in the context of MSK disorders. Competence can be described as what individuals know or can do in terms of knowledge, skills and attitude, whereas capability is the extent to which individuals can adapt to change, generate new knowledge and continually improve their performance [16]. The relationship between these two terms has been described as follows:

A competency […] is the capability to apply or use a set of related knowledge, skills, and abilities required to successfully perform ‘critical work functions’ or tasks in a defined work setting [17].

Our aim was to support service transformation by developing a nationally agreed core capabilities framework for first point of contact practitioners working with people with MSK conditions.

## Methods

**Fig. 1:**
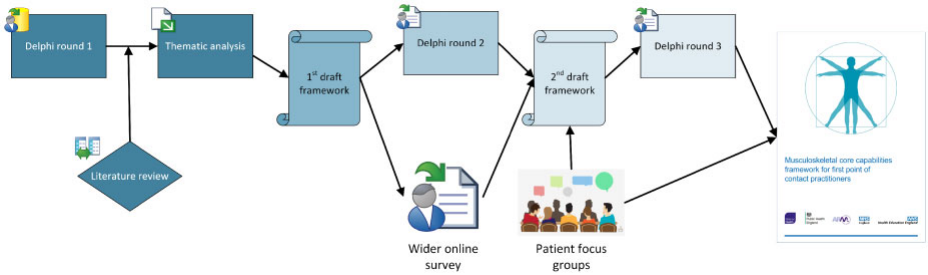
The framework development process

We used a multifaceted process to develop the framework, which was coordinated by a central project management group (Fig. 1). The project management group sought to represent a wide range of expert opinions through representatives from key stakeholder organizations, including Arthritis and Musculoskeletal Alliance (ARMA) and its members, Health Education England, NHS England, Public Health England, Skills for Health, professional bodies and higher education institutions (see Table 1). Ethical approval was obtained from the Faculty of Medicine & Health Research Ethics Committee, University of Leeds (MREC16-009).

**Table 1:** Organizations represented in the project management group

Arthritis Action
Arthritis and Musculoskeletal Alliance
British Society of Rheumatology
Chartered Society of Physiotherapy
Health Education England
Institute of Osteopathy
National School of Occupational Health
NHS England
Public Health England
Royal College of General Practitioners
Royal Cornwall Hospitals NHS Trust
Skills for Health
University of Central Lancashire
University of Exeter
University of Leeds
University of Salford

We sought to ensure that we built the framework around the needs of people with MSK conditions and that it maintained high levels of face validity with this group of key stakeholders. To this end, we organized four focus groups across England to explore what patients want from their initial consultation. The findings from that study are reported separately (Jo Erwin *et al.*, unpublished results). Furthermore, the project management group combined outputs from the different elements of the project and ensured that the final framework maintained face validity with different stakeholders to facilitate its implementation within services.

A key part of the framework development process was a modified three-round Delphi technique, which we selected owing to its constructivist nature of collating expert opinion and ability to build consensus amongst diverse stakeholders. We followed the recommendations for the conduct and reporting of Delphi studies (CREDES) [18]. We used a purposive sampling approach to recruit participants to the expert panel, who were nominated through 18 national professional and patient organizations (Table 2). The professional affiliations of participants in the three Delphi surveys can be seen in Table 3. Participants were presented with a participant information document, which included a consent form and advised that completion of the survey would constitute agreement to participate.

**Table 2:** Organizations that nominated expert representatives as participants in the Delphi survey

British Association of Prothetists and Orthotists
British Health Professionals in Rheumatology
British Institute of Musculoskeletal Medicine/Faculty of Sport and Exercise Medicine
British Orthopaedic Association
British Society for Rheumatology
Chartered Society of Physiotherapy
College of Paramedics
College of Podiatry
Health Education England
MSK: UK
National Health Service England
National Rheumatoid Arthritis Society
Primary Care Rheumatology Society
Royal College of General Practitioners
Royal College of Nursing
Royal College of Occupational Therapists
Royal Pharmaceutical Society

**Table 3:** Professional affiliations of participants in the three Delphi surveys

Round one (41 participants)	Round two (37 participants)	Round three (27 participants)
General practitioner MSK physician MSK service user NHS England Nurse Occupational therapist Orthopaedic surgeon Orthotist Paramedic Pharmacist Physiotherapist Podiatrist Public health medicine consultant Rheumatologist Senior strategy manager Sport and exercise medicine consultant Strategic health lead	General practitioner MSK physician Nurse Occupational therapist Orthopaedic surgeon Orthotist Paramedic Pharmacist Physiotherapist Podiatrist Public health medicine consultant Rheumatologist Sport and exercise medicine consultant	General practitioner MSK physician Nurse Orthopaedic surgeon Orthotist Pharmacist Physiotherapist Podiatrist Public health medicine consultant Sport and exercise medicine consultant

MSK: musculoskeletal; NHS: National Health Service.

The first round of the Delphi survey contained an open question, in which participants were asked to describe the capabilities required for competent clinical practice within MSK care. The expert panellists were also asked to provide information about any existing frameworks they already used. We carried out a search to identify additional frameworks and literature relevant to MSK practice in England. To explore the data, we used a theoretical approach and latent thematic analysis [18]. The units of analysis included the Delphi round one responses and the literature identified. Analyst triangulation was conducted in two ways. Firstly, data from the Delphi round one was analysed separately by two researchers (K.C.-L. and M.B.). Secondly, we used the emerging themes to inform the analysis of the existing frameworks identified in the literature search. The project management group then combined these analytical outputs to make an initial draft framework. For round two, we circulated this draft to the expert panel, who rated their agreement with each item on a 10-point Likert scale, where one represented not important at all and 10 represented extremely important.

Recognizing that nominated expert clinicians/practitioners can hold different views from front-line clinicians working in primary care [19], we also launched a wider online survey at this stage. This was to ensure that we captured the opinions of community-based clinicians, because previous research by Erwin *et al.* [20] highlighted that competencies put forward by a panel of national experts may be too detailed. Information about how to participate in this wider online survey was circulated through participating organizations and their networks. This survey sought feedback from a diverse range of practitioners wanting to provide comments or feedback, and participants were asked to rate their level of agreement with the capabilities and outcomes on a five-point Likert scale (agree, partly agree, undecided, partly disagree and disagree). This survey differed in structure from the Delphi survey, in that we asked participants only to first rate their level of agreement with each capability, and then their agreement with the set of the higher-level key outcomes (as opposed to each individual key outcome). We then developed the next draft framework by combining the results from round two of the Delphi survey with this wider online survey.

The third and final Delphi round asked the panellists to rate their agreement on the same 10-point Likert scale as used in round two. The expert panel also had opportunity to provide written feedback for each capability. When circulating the draft framework for round three of the Delphi survey, we offered the following information to participants for the description of professional values and behaviours, for the MSK underpinning knowledge and skills and for each capability:

a brief summary of the comments provided in round two to inform participants about the context for the development between rounds two and three; the group median of responses for round two; the interquartile range of the distribution of responses; and each participant’s round two rating, to show how they rated each capability in round two and enable a comparison of their rating with the rating by rest of the participants.

## Results

Eighteen national organizations nominated participants to round one of the Delphi survey, creating a multi-professional group of 41 expert participants. A list of participating organizations is provided in Table 2, and Table 3 shows the range of professional affiliations of participants. Combining the qualitative data from round one with the literature review produced the draft framework for round two, which comprised 14 capabilities grouped in four domains, and the capabilities included a total of 142 outcomes (see Supplementary Material, available at *Rheumatology Advances in Practice* online, section Draft framework for round two of the Delphi survey, available at *Rheumatology Advances in Practice o*nline). In addition to the capabilities and outcomes, we developed descriptions of professional values and behaviours and MSK underpinning knowledge and skills. It was considered that although these descriptors were developed in the same way as the rest of the framework, they underpin the capabilities without themselves constituting core capabilities. These areas underpin all the capabilities and are fundamental to the ability of a practitioner to demonstrate the outcomes. The literature included in the qualitative analysis can be found in Appendix 5 (p. 37) of the final framework [21].

Round two of the Delphi survey was completed by 37 participants (a 90% response rate), and their ratings (median values and interquartile ranges) for each capability are presented in Table 4. We received 90 responses to the wider online survey. Table 5 outlines the professional backgrounds of the people who participated in the wider online survey.

**Table 4:** Median values and interquartile ranges for each capability in Round 2 (D2) and Round 3 (D3) of the Delphi survey

	Median		Interquartile range	
	D2	D3	D2	D3
Domain A. Person-centred approaches				
Capability 1. Communication	10	10	9–10	10–10
Capability 2. Person-centred care	10	10	8–10	9.5–10
Domain B. Assessment, investigation and diagnosis				
Capability 3. History-taking	10	10	10–10	10–10
Capability 4. Physical assessment	10	10	10–10	10–10
Capability 5. Investigations and diagnosis	10	10	10–10	10–10
Domain C. Condition management, interventions and prevention				
Capability 6. Prevention and lifestyle interventions	10	10	8–10	9–10
Capability 7. Self-management and behaviour change	10	10	8–10	10–10
Capability 8. Pharmacotherapy	10	10	8–10	9–10
Capability 9. Injection therapy	10	9	6–10	6.5–10
Capability 10. Surgical interventions	10	9	7–10	7–10
Capability 11. Rehabilitative interventions	10	10	8–10	9–10
Capability 12. Interventions and care planning	10	10	9–10	9–10
Capability 13. Referrals and collaborative working	10	10	9–10	10–10
Domain D. Service and professional development				
Capability 14. Evidence-based practice and service development	10	10	8–10	9.5–10

**Table 5:** Professional affiliations of the people who participated in the wider online survey

Accident and emergency consultant	1
Chiropractor	1
Consultant orthopaedic surgeon	1
Consultant rheumatologist	1
General practitioner	3
Musculoskeletal physician/doctor	6
Nurse	5
Orthotist	1
Occupational therapist	1
Physiotherapist	50
Podiatrist	6
Unknown	14
Total	90

We used the ratings and comments from round two of the Delphi survey and the wider online survey to refine the framework into its next draft form (see Supplementary Material, available at *Rheumatology Advances in Practice* online, section Draft framework for round three of the Delphi survey, available *at Rheumatology Advances in Practice* online). A recurrent theme in the comments we received in round two described a significant overlap and some duplication across the framework. As a result, we rephrased or combined several statements and sections, and the draft framework still comprised four domains and 14 capabilities but with 103 outcomes.

Round three of the Delphi survey was completed by 27 participants (a 73% response rate). The median level of agreement for all 103 outcomes was at least nine in the final round. Table 5 shows the median values and interquartile ranges for each of the 14 capabilities for rounds two and three.

The project management group combined the results of round three, including ratings and comments, to finalize the framework. The final framework contains 105 outcomes within 14 capabilities, separated into four domains. The development from 103 (round three of the Delphi survey) to 105 (final framework) outcomes was a result of combining four outcomes into one (Capability 2e) and adding five suggested outcomes (Capability 6d, 6e, 7 h, 8f and 11d). This development was in response to comments from participants in round three of the Delphi survey.

The domains and capabilities can be seen in Table 5, and the full framework document can be accessed from http://www.skillsforhealth.org.uk/services/item/574-musculoskeletal-core-skills-framework.

## Discussion

This capability framework has been developed with representatives from the whole MSK community in England. It provides clarity on the expected standards, knowledge, skills and behaviours of practitioners dealing with people who have MSK conditions at the first point of contact. By making better decisions early in the patient journey, it is likely that patient care and outcomes will improve. Having this MSK core capabilities framework can help to ensure that the health professionals who provide care for people with MSK conditions are prepared to manage this group of patients effectively. The framework recognizes the different levels of capabilities within the scope of practice of the different professions and emphasizes the importance of team working and person-centred care. Some health professionals will already be working in accordance with the capabilities, fully or partly, and the framework offers guidelines for continuing professional development to reach a standard of safe, effective and consistent practice. The framework offers opportunities to develop training and development of the MSK workforce and to increase the number of practitioners from different professions who can undertake the first contact role.

This framework offers clear definitions for clinicians, employers, regulators, commissioners and education providers of the capabilities required for the delivery of high-quality MSK care. The skills, values and behaviours needed to offer this care are manifold, and the breadth of the domains, capabilities and outcomes reflects this. The scope of the framework is MSK focused, yet wide, and includes facets from person-centred care to pharmacotherapy; from being able to engage with the impact of persistent pain and disability to having the skills to address individuals’ fears about medications. This wide scope combined with the very high level of agreement we recorded in the Delphi process underlines the need for a biopsychosocial approach to the effective management of MSK conditions. The emphasis on a person-centred approach is underlined by the inclusion of the Patient journey section in the framework, developed through four focus groups organized across England to explore what patients want from their initial consultation (submitted for publication).

A clear outline of competencies is fundamental to health-care education curricula and can shape the attributes of graduates by informing learning outcomes and assessment thresholds. To equip health professionals with new capabilities requires strategies for both the current and future workforce. Different health disciplines have different sets of competencies and capabilities determined by their respective accrediting organizations. Musculoskeletal care is but one of many areas within these disciplines, and we recognize the challenges associated with mapping curricula and professional development training with multiple frameworks. The capabilities included in the framework can be acquired at both pre- and post-graduate levels.

Competency-based education (CBE) is an educational delivery method that has been suggested as a way of delivering quality health care through competent health professionals [22]. The principles of CBE include a focus on outcomes, emphasis on abilities and promotion of learner-centredness, and most health professions have moved towards CBE as part of a shift from a training to an education focus [23–25]. However, some authors have argued that this shift has caused profession-specific clinical skills to take a back seat to other priorities, and that sets of competencies can be vague and fail to distinguish between professions [26]. We argue that the capabilities in the framework are not profession specific but have the patient at the centre. The way in which we have set out what each first point of contact MSK practitioner should be able to do helps education and training providers to design and deliver appropriate content. This aligns well with a CBE approach to workforce development.

We acknowledge that each profession will have a different starting point, determined by clinical training and scope of practice. Some practitioners may need to develop additional skills to meet all of the capabilities, whereas others might already be working in accordance with them. The intention of the framework is to ensure that first point of contact practitioners are skilled in diagnosis, prevention, supported self-management advice, early intervention and, where needed, onwards referral, for those presenting with an MSK condition. This focus differs from that described in the multi-professional framework for advanced clinical practice in England, which describes the capabilities required to work at a level of practice characterized by a high degree of autonomy and complex decision making, and which is underpinned by a master’s level award or equivalent [27]. In other words, the frameworks complement each other, with the latter building on the former.

The NHS Long Term Plan [11] and new NHS England GP Contract [12] seek to ensure that patients have direct access to MSK FCPs, and the framework enables commissioners of MSK services to specify the standards for clinical care by setting out clear expectations about what first point of contact practitioners are able to do for people presenting with undiagnosed MSK conditions. Service providers and clinical managers can use the framework to evaluate service needs and put development plans in place, to help ensure that clinical practice is up to date, safe and effective. On an individual level, practitioners and teams can identify training needs by comparing current with required capabilities. Future studies should seek to evaluate the FCP role and the impact of this framework.

There are both limitations and strengths regarding the development and scope of the framework. Developing a framework of this nature is an inherently complex process, and we have sought to offer transparency on the development process through this publication in a way that has not always been achieved for other frameworks. This framework is limited to an adult population and does not outline the specialist knowledge and skills required for those managing paediatric MSK presentations. A key strength of our study is the modified Delphi technique, including a wide range of stakeholders, to achieve a national consensus about a contemporary set of MSK core capabilities for first point of contact practitioners. The CREDES standard explicitly states that the Delphi technique is flexible and can be adjusted for the specific objectives of a study [18]. The Delphi technique is commonly used in a modified form. In their systematic review, Boulkedid *et al.* [28] found that 49 (63%) of their identified Delphi studies were modified versions of this method. Although the purposive participant selection method may not adequately represent the full spectrum of views across all the relevant professions, but we sought to mitigate this by including a wider online survey. The range of professional affiliations of participants in the three rounds of the Delphi surveys and the wider online survey can be seen in Tables 3 and 5, respectively, and this heterogeneous group ensured that diverse and varied perspectives were included. Attrition is commonplace in most longitudinal studies regardless of design, and our Delphi was no exception. We had a 90% response rate for round two and 73% for the final round, which is comparable to figures reported in a systematic review of the Delphi method [28].

Although the patient voice was represented in the framework development process, we acknowledge that this could have been more substantial in the Delphi itself. Although we asked for organizations to nominate multiple patient representatives, only one completed the first round, and we were unable to introduce more patients to subsequent rounds in line with the Delphi methodology. However, the project management group included a service user, and the patient journey section, in its entirety, was based on four focus groups recruited from service users.

The systematic review undertaken during the development of the CREDES guidelines found that the number of rounds in Delphi studies ranged from one to five and recommended at least two rounds [18]. A literature review of consensus measurement in Delphi studies found that a general standard of how to measure this has not been established [29]. Owing to the pragmatic nature of this study, we did not establish an *a priori* definition of consensus for the first two rounds of the Delphi study. We made this decision in the context of the wide scope of the framework, including geographical span (England), diverse range of health-care professions and relevant capabilities. For Delphi rounds two and three, we decided a cut-off point of nine as a median level of agreement. Boulkedid *et al.* [28] found a that the method used to define consensus varied across studies, and our determined level is greater than that typically used in Delphi studies.

The promotion of the framework capabilities might encourage a change in behaviour in the current and future clinician workforce, including primary care doctors, specialist nurses, clinical pharmacists and allied health professionals. The capabilities are relevant to a range of settings and types of service provision, including, but not limited to, primary care, community care and occupational health.

## Conclusion

The framework provides a standard structure and language across professions, thereby promoting greater consistency and portability of MSK core capabilities. The framework enables service commissioners to specify minimum standards of clinical care, and service providers to demonstrate that staff meet the standards of the nationally recognized framework or have developmental plans in place to do so. It also allows education and training providers to design programmes and curricula that meet the needs of future FCPs, and practitioners to map existing skills and learning needs against nationally agreed role requirements.

The framework describes the capabilities required for practitioners working in first point of contact roles for people with MSK conditions. Despite the diverse profile of participants, reflecting a broad range of professional roles, levels of agreement were high. The framework is now being disseminated by Health Education England, NHS England and Skills for Health across England and is being incorporated into practice and service redesign.

## Supplementary Material

rkz036_Supplementary_Materials
