# Supplementary material for: Developing a national musculoskeletal core capabilities framework for first point of contact practitioners
Source: Rheumatol Adv Pract. 2019 Sep 20;3(2):rkz036. doi: 10.1093/rap/rkz036 (PMC6799852; doi:10.1093/rap/rkz036)
Supplement: rkz036_Supplementary_Materials [file rkz036_supplementary_materials.docx]

**SUPPLEMENTARY MATERIAL**

*Draft framework for round two of the Delphi survey*

This survey seeks your views on the draft **Musculoskeletal Core Capabilities Framework** for first point of contact clinicians. By completing the survey you are giving consent for the data to be used to further develop this framework.

**Scope of the framework**

The scope of this framework covers the **core capabilities** for clinicians likely to be the first point of contact for people presenting with undiagnosed musculoskeletal conditions.  The expectation is that first point of contact will enable the development and implementation of a management plan, with onward referral only when required. Therefore, the first point of contact clinician will assess, diagnose, develop and agree a management plan, offer initial treatment advice (including treatment if the pathway allows) and discharge.

First point of contact clinicians include health professionals such as a GP, physiotherapist, podiatrist, occupational therapist and other health professionals able to apply the capabilities in their practice. The framework describes core capabilities to meet the needs of people with MSK that are common and transferable across different types of service provision and settings, including but not limited to; primary care, emergency medicine and occupational health.

The framework also provides a focus on the workforce capability to support shared decision-making, care and support planning as well as prevention, self-management, fitness for work and meaningful behaviour change.  As such, there are synergies with other frameworks such as the recently launched Person-Centred Approaches framework (Health Education England & Skills for Health 2017).

As the scope of this framework is **core capabilities**, it follows that many professionals will have additional knowledge and skills beyond the common core.  In particular, specialist skills and knowledge for those managing MSK conditions that present high levels of complexity and high levels of uncertainty and risk (e.g. relating to patients with multiple long-term conditions and multiple healthcare needs) are outside the scope of this framework.

**What is outside the scope of this framework?**

As the scope of this framework is **core capabilities**, it follows that many professionals will have additional knowledge and skills beyond the common core.  In particular, specialist skills and knowledge for those managing MSK conditions that present high levels of complexity and high levels of uncertainty and risk (e.g. relating to patients with multiple long-term conditions and multiple healthcare needs) are outside the scope of this framework. In addition, this framework is not focused on those who deliver treatment interventions for previously diagnosed conditions i.e. those who are not first point of contact.

The expectation is that subsequent frameworks for other specialist roles will build on the structure and capabilities presented here so that the commonality facilitates understanding, communication, and career development.

**Structure of the framework**

The framework begins with a description of professional values, behaviours, and knowledge, which underpin all capabilities in this framework.

The draft framework comprises 14 capabilities grouped in 4 domains, and each capability has a range of key outcomes.

## Professional Values and Behaviours

The following professional values and behaviours focus on the expectations of people with MSK conditions and their carers.

The MSK first point of contact practitioner will demonstrate safe, effective, autonomous and reflective practice utilising appropriate clinical guidelines while accepting accountability for outcomes of their interventions.  They will work effectively as part of a team; either as a leader or as a team member, utilising their skills and attributes of the team in the best interests of the patient and service.

They will work ethically, and will incorporate a critical approach to ethical uncertainty, working actively with others to resolve ethical conflict and in accordance with existing legal, regulatory and ethical requirements or codes relevant to professional bodies and employers

- Be interested in, and want to understand and respond to people’s perspective, their preferences and what is important to them and their carers.
- Value and acknowledge the experience and expertise of people, their carers and support networks.
- Recognise the impact and frustrations that painful, often chronic, conditions, can have on both the person with an MSK condition and the practitioner.
- Understand the practitioner’s role can be more than simply fixing the issues raised - supporting and enabling people to live meaningful lives is important, whether or not cure or resolution is possible.
- Ensure integrated current and future care, support and treatment, through working in partnership with people, teams, communities and organisations, understanding and respecting each other’s roles and competencies.
- Value collaborative involvement and co-production with people with MSK to improve the person-centred design and quality of services.
- Enable and participate in research to advance the development of MSK knowledge and practice.
- Possess an appropriate range of clinical knowledge and skills that enable the practitioner to objectively examine, interpret findings, develop working and differential diagnoses and develop and implement a management plan.

## MSK Underpinning Knowledge

The capabilities described in this framework assume an underpinning knowledge essential to promote musculoskeletal health, and to diagnose and manage people with musculoskeletal conditions.  This includes a comprehensive understanding of the normal structure and function of the MSK system and conditions that can affect musculoskeletal health.

The MSK practitioner will also demonstrate a deep and systematic understanding of the biological and psychosocial sciences that are applicable to the MSK system, interrelated bio-psychosocial systems and relevant disciplines and how these impact on people and society in the framework of WHO ICF, and the prevention and management of MSK conditions.

The practitioner will be able to clinically apply such knowledge and understanding in the best interests of the patient, specifically in relation to the context of the delivery of the first level of care and within their professional scope of practice.

- The practitioner will understand the sciences associated with musculoskeletal health such as structure and function of bone, joints, muscle, connective tissue and disease processes relevant to supporting the management of MSK problems.
- The practitioner will understand the physiology, cell biology and pathology relating to musculoskeletal conditions which include the ageing process, injury and disease states and repair of musculoskeletal tissues such as bone, cartilage, synovium, muscle and enthesis.
- The practitioner will know of the common MSK conditions and be aware of less common and rare MSK conditions. An indicative (not exhaustive) list of common MSK problems will include: musculoskeletal injury; joint pain – poly, mono, and periarticular; back pain; regional pain or stiffness; generalised pain or stiffness; decreased or loss of motion or weakness; altered sensation; deformity; mass.
- The practitioner will know which MSK conditions are emergencies or urgencies. (Red flags include open fractures, fractures associated with nerve or vascular compromise, cauda equina syndrome, joint infection, soft tissue infection, bone infection and temporal arteritis. Urgencies include inflammatory joint and spine diseases).

## Domain A. Person-Centred Approaches

MSK practitioners will possess and develop interpersonal and communication skills that are necessary and appropriate for effective communication within the clinical environments in which they work. This will include understanding the needs and concerns of people with MSK conditions, using appropriate language and media in the best interests of patients and carers, and in communication with colleagues and other health and care professionals.

MSK practitioners will enable people with MSK conditions to talk about their concerns and priorities, to know about their options and what is known of the risks, benefits and consequences of all reasonable courses of action.  The individual’s specific needs, wants and circumstances should guide the care and treatment offered.  The individual must be recognised as the expert in their own life and be empowered and supported to retain control.

## Capability 1 - Communication

The practitioner will:

- Be aware of own values, beliefs, prejudices, assumptions and stereotypes when working with people
- Understand the value of really listening as an active process
- Understand communication as a two-way process
- Understand the impact of conversations and different verbal and non-verbal communication styles
- Know how to meet the communication and language needs, wishes and preferences of individuals
- Be able to discuss issues in a way that avoids jargon
- Understand the concerns of the person about their current situation and fears of the future, in particular the fear of future disability
- Understand the reasons for positive terms such as ‘wear, flare and repair’ and avoid unhelpful terms like ‘it’s because of your age’, ‘crumbly spine’ and ‘ruptured disc’
- Be able to communicate effectively with other professionals and colleagues using a variety of media (e.g. verbal, written and digital)
- Understand the importance of respecting the knowledge and expertise of other professionals and colleagues
- Be able to confidently demonstrate core communication skills for relationship building and information gathering.

## Capability 2 - Person-centred care

The practitioner will:

- Understand the significance of a person’s background, culture, values and experiences when providing their care and that each person is an expert in their own life
- Understand the impact of chronic pain and disability on individuals’ lives, including the impact on relationships, self-esteem and ability to participate in what they need and want to do including paid and unpaid work
- Be aware of the burden of treatment for patients with long-term musculoskeletal conditions, many of whom will be attending regularly for appointments about other long-term conditions
- Understand the importance of restoring and maintaining function and independence as a clinical outcome
- Understand that reducing pain and improving quality of life rather than achieving a complete cure could be the goal of treatment
- Be able to assess individuals’ levels of activation and health literacy and modify conversations accordingly
- i - Be able to enable a person to make decisions by understanding the priorities and outcomes that are important to them

ii - Be able to enable a person to make decisions by explaining in non-technical language all the available options (including the option of doing nothing)

iii - Be able to enable a person to make decisions by exploring with them the risks, benefits and consequences of each option and discussing what these mean to the person in the context of their life and goals

iv - Be able to enable a person to make decisions by supporting them to be able make the decision and / or agreeing together the way forward

- Be able to signpost appropriately and effectively to information and support
- Be able to respond to individuals’ communication and information needs and support the use of accessible information where appropriate

## Domain B. Assessment, Investigation and Diagnosis of MSK Problems

The MSK first point of contact practitioner will be able to conduct clinical assessment and develop a differential diagnosis – which may require appropriate investigations and tests.

**Cognitive Skills**

The MSK first point of contact practitioner will demonstrate conceptualisation and critical thinking in respect of received wisdom. They will have skills that will enable them to problem solve and research areas of practice and to critically evaluate outcomes of interventions.

They will analyse and synthesise information, particularly in relation to unfamiliar contexts and presentations where information maybe contradictory. They will undertake appropriate evaluations and appraisals of interventions.

**Performance & Practice**

The MSK first point of contact practitioner will demonstrate safe, effective, autonomous and reflective practice drawing on innovation, and established best practice. They will work effectively as part of a team; either as a leader or as a team member, utilising their skills and attributes of the team in the best interests of the patient and service.

They will work ethically and will incorporate a critical approach to ethical uncertainty, and will work actively with others to resolve ethical conflict.

## Capability 3 - History taking

The practitioner will:

- Be able to carry out a comprehensive subjective assessment based on knowledge of the characteristics of musculoskeletal conditions, including presenting complaint; past medical history; medication; relevant social, leisure and employment factors
- Be able to assess the impact on the individual due to symptoms including impairment of function, limitation of activities and restriction of participation including work
- Be able to explore factors affecting people’s ability to participate in life situations such as work. This should include ‘yellow flags’ and perceptions about the relationship between work and health, sometimes referred to as ‘blue flags’ and ‘black flags’
- Be able to recognise and resolve conflicting information and complexity based on enquiry and clinical reasoning skills
- Be able to use effective questioning and communication skills to effectively gather information
- Understand that musculoskeletal symptoms can be features of non-MSK conditions, sometimes due to serious pathologies
- Understand that musculoskeletal symptoms can be compounded by psychological factors and mental health issues
- Understand the potential impact of life-style factors such as smoking, alcohol and illicit drug use
- Be able to take a history of acute and chronic pain and assess its effects on the person
- Be aware of the symptoms that are associated with MSK problems such as pain, stiffness, weakness, fatigue, limitation of activities and restriction of participation
- Be able to explore the perceptions, ideas or beliefs the patient has about their condition and whether these may be acting as barriers to recovery or return to usual activity or work
- Be able to concisely and accurately record clinical documentation

**Capability 4 - Physical assessment**

The practitioner will:

- Know how to obtain patient consent to physical examination
- Understand the importance of maintaining the privacy, dignity and comfort of the patient as far as practicable
- Be able to comply with infection prevention and control procedures
- Understand the need to adapt practice to meet the needs of different groups and individuals, including the need for chaperones where appropriate
- Be able to conduct a GALS initial screening assessment
- Be able to appropriately, systematically and effectively apply a range of assessment techniques and know their validity, reliability and specificity
- Be able to carry out observation and functional assessment relevant to the presentation to identify and describe normality and abnormality, e.g. palpate joints; recognise joint inflammation; recognise bone / joint trauma; recognise loss of function – weakness, restricted movement, deformity and disability, ability to perform usual tasks or occupation; recognise patterns of joint pain
- Be able to resolve conflicting information, test results and complexity
- Understand the need for clinical assessment of integumentary, cardiovascular, and respiratory systems
- Be able to concisely and accurately record clinical documentation

**Capability 5 - Investigations and diagnosis**

The practitioner will:

- Be able to characterise and identify the MSK problem from the clinical assessment
- b) Be able to recognise different patterns / syndromes including: regional pain or stiffness; joint pain – mono, poly, periarticular; generalised pain or stiffness; neck pain; back pain; bone pain; muscle pain, stiffness or weakness; systematic problems – extra-skeletal problems; musculoskeletal injury;
- c) Be able to assess the importance and meaning of presenting features – the following is an indicative list: pain: nature, location, severity, history of trauma; variation of symptoms over time; symptoms which help distinguish inflammatory from non-inflammatory conditions; loss of function – weakness, restricted movement, deformity and disability, ability to perform usual tasks or occupation; systemic manifestations of rheumatic disease
- Be able to identify 'red flags' (and serious pathology which may be masquerading as a MSK condition), and make urgent onwards referral
- Be able to identify risk factors for severity or impact, sometimes referred to as ‘yellow flags’ and be aware of tools that can be used to stratify those at risk of progression to long-term pain and disability
- Be able to diagnose common problems that can usually be managed in primary care, including regional soft-tissue problems and regional pain syndromes such as osteoarthritis, back pain and fibromyalgia
- Be able to recognise conditions which may benefit from early referral to secondary care
- Understand that early diagnosis of inflammatory arthritis has a major impact on long term outcomes and the importance of speedy referral when there is clinical suspicion
- Be able to recognise how musculoskeletal problems may be a manifestation of injury not only from trauma but also abuse
- Be able to order or refer for appropriate investigative tests to aid diagnosis and assessment and to understand / interpret the results
- Understand indications and limitations of blood tests for diagnosing and assessing musculoskeletal conditions
- Understand indications and limitations of plain radiography, ultrasound, CT and MR scan including the use of decision-making tools
- Understand the effective use of scarce, expensive or potentially harmful investigations

**Domain C. Condition Management, Interventions and Prevention**

The MSK first point of contact practitioner will support and encourage self-management and changes in behaviour that will have a positive impact on the health and wellbeing of individuals, communities and populations. They will advise on interventions and therapies and enable the development and implementation of management plans, often working in collaboration with colleagues and other healthcare professionals.

**Capability 6 - Prevention and lifestyle interventions**

The practitioner will:

- Understand the impact that a range of social, economic, and environmental factors can have on outcomes for people with MSK conditions, their carers and their circles of support
- Understand the importance of social networks and communities for individuals and their carers
- Understand the importance of physical activity in promoting musculoskeletal health and what people with MSK conditions can and should do
- Understand the effects of smoking, obesity and inactivity on MSK health and conditions
- Understand the effects of injuries on MSK health
- Understand how work-related MSK conditions may be prevented through acting on effective risk assessments and the provision of safe working conditions
- Be able to act on day to day interactions with people to encourage changes in behaviour that will have a positive impact on the health and wellbeing of individuals, communities and populations, i.e. ‘Making Every Contact Count’
- Be able to facilitate behaviour change using evidence based approaches such as motivational interviewing and supporting self-management
- Be able to measure, monitor and report population health and wellbeing, health needs, risk and inequalities and use of services
- Be able to promote population and community health and wellbeing, addressing the wider determinants of health and health inequalities
- Be able to work collaboratively across agencies and boundaries to improve health outcomes and reduce health inequalities

**Capability 7 - Self-management and behaviour change**

The practitioner will:

- Understand the principles of behaviour change
- Evaluate established health coaching tools and techniques
- Understand ‘patient activation’ and its role in supporting individuals to self-manage
- Understand the importance of supporting the individual to fulfil their role in their management plan
- Be able to support people by exploring consequences of actions and not taking actions, e.g. appropriate use of exercise and medication
- Be able to support individuals’ self-management and behaviour change to optimise their physical activity, mobility, fulfilment of personal goals and independence
- Be able to promote patients’ self-management using a range of methods including coaching skills, option grids and shared decision-making
- Enable individuals to get the best from conversations by supporting and encouraging them to ask the right questions
- Be able to advise individuals on specific aspects of self-management, e.g. goal setting, pacing and exercise programmes, relevant to particular conditions
- Be able to identify risk factors for persistence and impact of MSK conditions ('yellow flags') and manage psychosocial implications
- Understand that MSK conditions are often coupled with mental health issues and know how to access psychology and counselling services
- Be aware of the expected natural history and prognosis for MSK conditions
- Know the sources of relevant local or national self-help guidance, information and support

**Capability 8 – Pharmacotherapy**

The practitioner will:

- Know the most common medications used in MSK and pain disorders, including analgesics, non-steroidal anti-inflammatory drugs, corticosteroids, drugs used in treating patients with metabolic bone diseases, gout, and in the management of patients with chronic pain
- Understand the indications, contraindications, expected benefits and limitations, side effects and drug interactions of commonly used drugs
- Know of the rationale and features of disease-modifying anti-rheumatic drugs and biological agents used for inflammatory arthritis
- Know how to access appropriate prescriptions within current MSK primary care guidelines
- Understand public health issues in prescribing including the potential for the misuse of drugs
- Understand the importance of reviewing people’s response to medication

**Capability 9 - Injection therapy**

The practitioner will:

- Understand role of joint injections and the evidence base for injections of drugs in musculoskeletal practice
- Be able to determine the possible benefit of injection for the individual’s problem and inform the patient impartially on the potential benefits and disadvantages of the procedure
- Be able to allay the patient’s unreasonable fears and beliefs concerning injections
- Where giving injections is applicable to professional scope of practice, the practitioner will:
- Understand indications, contra-indications for joint and soft tissue injections carried out in musculoskeletal practice
- Understand side effects and interactions of commonly used drugs for musculoskeletal injection
- Be able to ensure the patient’s comfort during the injection and to minimise the discomfort of the injection itself
- Be able to carry out the procedure with techniques to ensure accuracy and minimum possibility of infection
- Be able to advise patients and carers (where relevant) about important after effects of injection including interactions, and the possibility of infection

**Capability 10 - Surgical interventions**

The practitioner will:

- Be aware of the most common operations for musculoskeletal conditions. This includes arthroscopy, arthroplasty of hip and knee, back surgery (discectomy, spinal fusion), amputation, synovectomy, osteotomy, arthrodesis, and nerve / carpal tunnel decompression
- Understand role of surgical procedures
- Be able to determine the possible benefit of surgery for the individual’s problem and inform the patient impartially on the potential benefits and disadvantages of the procedure
- Be able to allay the patient’s unreasonable fears and beliefs and to clarify or explain the opinions provided by other health professionals concerning surgery

**Capability 11 - Rehabilitative interventions**

The practitioner will:

- Understand the role and benefits of rehabilitation of MSK conditions, the different modalities available to restore and maintain function and participation and when they should be considered
- Be able to determine the possible benefit of rehabilitative interventions for the individual’s problem and inform the patient impartially on the potential benefits and disadvantages of the interventions
- Understand the roles of specialist rehabilitation practitioners such as occupational health practitioners and other rehabilitation support staff
- Understand that the longer the period of absence from work, the higher the risks of prolonged absence, reduced return to work and subsequent loss of employment
- Be able to assist patients in identifying and using strategies to address work instability and improve work retention
- Be able to make recommendations to employers regarding fitness to work, e.g. through the appropriate use of a fit note

**Capability 12 - Interventions and care planning**

The practitioner will:

- Understand management strategies for common MSK problems, including but not limited to: musculoskeletal injury; joint pain – poly, mono, and periarticular; back pain; regional pain or stiffness; generalised pain or stiffness; decrease or loss of motion or weakness; altered sensation; deformity; mass
- Be able to develop a management plan that takes into account the needs and wishes of the patient and local availability of services
- Understand the links between prolonged MSK symptoms and reduced mental wellbeing and have knowledge of sources of mental health support.
- Be able to provide acute injury management of bone, joint and soft tissue, e.g. rest, ice, pressure, compression or elevation.
- Be able to manage acute systemic inflammatory conditions such as gout and polymyalgia rheumatica
- Be able to provide advice on pharmacological and non pharmacological aspects of acute and chronic pain management
- Be able to prescribe a personalised exercise programme as part of rehabilitation
- Be able to make appropriate referrals to other health care professions and outside agencies when required
- Be able to provide basic rehabilitation advice including: graded return to normal activity; navigation to self-care resources; reassurance of the need for modified activity for a limited period of time
- Be able to identify when first-line intervention has been successful and discharge the patient

**Capability 13 - Referrals and collaborative working**

The practitioner will:

- Be able to understand and work within one’s personal and professional scope of practice and know how and when more specialist advice or support should be sought
- Understand the importance of effective inter-professional collaboration to optimise patient, or population care for many MSK problems
- Be able to communicate with and make appropriate referrals to other health care professions and outside agencies when required
- Know other services locally that an individual may benefit from as part of a person centred approach - including employment related, voluntary, counselling services and leisure facilities
- Know the roles played by all members of the multi-disciplinary team and the importance of integrated care
- Be able to contribute effectively to work undertaken as part of a multi-disciplinary team
- Be able to participate as an effective team member and understand the importance of effective team dynamics

**Domain D. Service Development**

The MSK first point of contact practitioner will support the development of MSK services through contributing to on-going research and development of evidence-based practice.  This will include evaluating the outcomes and impact of services and interventions.  When people with MSK conditions participate in research activities, this must be handled with due regard to the ethical issues involved.

The MSK first point of contact practitioner will also demonstrate reflective practice in personal evaluation and development. They will plan their own development in response to self-reflection, peer review and performance feedback.

**Capability 14 - Evidence-based practice and service development**

The practitioner will:

- Be aware of relevant national guidance (e.g. NICE guidelines) and potential problems in applying these guidelines based on local availability of services
- Understand the key role research plays within the NHS Constitution, as well as codes of conduct from regulatory and professional bodies
- Know of MSK conditions where specific guidelines for investigations, referral and management are in place
- Be aware of the range of evidence that informs planning, implementation and evaluation of MSK care
- Understand the need to monitor and evaluate the quality of practice and the value of contributing to the generation of data for quality assurance and improvement programmes
- Know the difference between audit, service evaluation and research and the differing regulatory frameworks around each
- Understand approaches to evaluating services and measuring impact, including input from individuals cared for and other stakeholders to inform ongoing improvement
- Be able to participate in audit programmes and research, including implementing change as part of the audit cycle (contributing or leading as appropriate to own role)
- Be aware of the potential effect on the health of patients where services are deficient e.g. frequent long waiting times
- Be able to check how changes in practice are affecting people who are being cared for and take corrective action as needed
- Understand the importance of involvement and co-production with patients and carers to improve the person-centred design and quality of services
- Be able to develop knowledge and competence through on-going education and review of the evidence base in relation to clinical practice
- Understand the importance of reflective practice and effective clinical supervision

**Supplementary Appendix B**

*Draft framework for round three of the Delphi survey*

Thank you for participating in the previous two rounds of this Delphi study to develop a **Musculoskeletal Core Capabilities Framework** for first point of contact clinicians. We now invite you to complete this survey to complete the third and final round.

Following the last round, we have used your ratings and comments to refine the framework into its current form. A recurrent theme in the comments we received in the last survey was that there was significant overlap and some duplication across the framework. As a result of this, several statements have been re-phrased or combined.

We would now like to find out how important you think it is for each of the items in the new draft to be included in the final framework.

The following additional information is provided for each section and capability in this round:

- A brief summary of the comments provided in Round 2 – this information is included to inform you about the context for the development of each section/capability between Round 2 and Round 3.
- The Group Median – the average value of the two numbers in the centre of the distribution of responses for round 2.
- The Interquartile Range - a measure of the spread of the distribution of responses, describing the middle 50% of the data. If there was a lot of disagreement within the group about a specific section, the interquartile range would be larger than if the group had similar views.
- Your Round 2 rating – this shows how you rated each section/capability in Round 2 and lets you compare your rating with the rest of the participants’ rating.

## Professional Values and Behaviours

**Summary of comments from Round 2:**

Respondents stressed the importance of this section being included, but suggested that it needed some revision in terms of vocabulary and terminology used.

**Delphi Round 2 median participant rating:** 10

**Interquartile range:** 10 to 10 (no difference between data in the mid-50% of the distribution)

**Your Delphi Round 2 rating:**

As registered health professionals, first contact practitioners do the following:

- Adhere to legal, regulatory and ethical requirements, professional codes, and employer protocols.
- Adopt a critical approach to ethical uncertainty and work with others to resolve ethical conflict.
- Demonstrate safe, effective, autonomous, reflective practice.
- Inform their practice and professional development and remain up-to-date with the best available evidence through the use of clinical guidelines and research findings.
- Are accountable for their decisions and actions and the outcomes of their interventions.
- Work effectively as part of a team, using their professional knowledge and skills, and drawing on those of their colleagues.
- Seek to meet individuals’ best interests and to optimise service delivery.

As more specific elements of their professionalism, first point of contact MSK practitioners:

- Seek and engage with individuals’ perspectives on their condition, their preferences for their care, and what is important to them and their carers in terms of treatment goals and outcomes.
- Value and acknowledge the experience and expertise of individuals, their carers and support networks.
- Use their clinical reasoning skills to undertake assessment of presenting problem, interpret findings, develop working and differential diagnoses, formulate, communicate, implement and evaluate management plans.
- Recognise the wider impact that painful, often persistent, conditions can have on individuals, their families and those close to them.
- Understand that their role is to support and enable individuals to lead meaningful lives, whether or not cure or resolution is possible.
- Ensure integrated care, support and treatment, through forward-planning, working in partnership with individuals, different professionals, teams, communities and organisations, and understanding, respecting and drawing on others’ roles and competence.
- Value collaborative involvement and engage people with MSK conditions to improve and co-produce person-centred quality services.
- Enable and participate in research to advance the development of MSK knowledge and practice.

## MSK underpinning knowledge and skills

**Summary of comments from Round 2:**

References to specific professional groups should be avoided; limited list of MSK conditions should be changed as it is unlikely to be an exhaustive list; some of this content is unnecessary as overlaps with existing curricula for various professions.

**Delphi Round 2 median participant rating:** 10

**Interquartile range:** 10 to 10 (no difference between data in the mid-50% of the distribution)

**Your Delphi Round 2 rating:**

- The capabilities set out in this framework require the clinical knowledge and skills to promote MSK health and to diagnose and manage the care of individuals with MSK conditions.
- First point of contact practitioners have a comprehensive understanding of the normal structure and function of the MSK system. They understand common MSK conditions and are aware of less common and rare MSK conditions.
- They understand the impact that MSK conditions have on individuals and on society in the context of the bio-psycho-social model and the framework of the World Health Organisation International Classification of Functioning, Disability and Health.
- They recognise and understand signals that indicate the need to refer individuals to specialist care and investigations, and when presenting symptoms or conditions require urgent or emergency intervention.
- They also have a strong understanding of health promotion and illness prevention, more specifically how to manage and prevent MSK conditions, and the skills to support individuals’ self-management and behaviour change.
- They have clinical-reasoning and problem-solving capabilities and critical self-awareness skills both to apply their knowledge and skills within their professional scope of practice, and to know when to seek advice and to make referrals to others to meet the best interests of the individuals they serve.

## Domain A. Person-Centred Approaches

MSK first point of contact practitioners have the interpersonal and communication skills to engage in effective, appropriate interactions with individuals, carers and colleagues in the clinical environments and roles in which they practise. They have the listening, information-processing and empathetic skills to ascertain, understand and respond to individuals’ needs and concerns. They use appropriate language and media, are sensitive to individual preferences and needs, and uphold and safeguard individuals’ interests.

Practitioners take account of individuals’ specific needs, wants and circumstances to guide the care and treatment they offer. They respect individuals’ expertise in their own life and condition, and empower and support them to retain control and to make choices that fit with their treatment goals.

MSK practitioners enable individuals to talk about their concerns and priorities relating to their MSK condition and its implications. They help individuals to understand their care options, sharing information on the risks, benefits, consequences, and potential outcomes in a clear, open way.

## Capability 1 - Communication

**Summary of comments from Round 2:**

There is a lot of overlap between the outcomes within this capability and with content in other sections; consider some revision in terms of vocabulary and terminology used; too much detail.

**Delphi Round 2 median participant rating:** 10

**Interquartile range:** 9 to 10

**Your Delphi Round 2 rating:**

The practitioner can do the following:

- Use a critical self-awareness of their own values, beliefs, prejudices, assumptions and stereotypes to mitigate the impact of these in how they interact with others.
- Listen to and communicate with others, recognising that both are an active, two-way process.
- Modify conversations to optimise individuals’ engagement and understanding, informed by assessing individuals’ levels of activation and health literacy.
- Adapt how they engage with others through using different verbal and non-verbal communication styles, and in ways that are responsive to individuals’ communication and language needs and preferences.
- Convey information and discuss issues in ways that avoid jargon, negative descriptors and assumptions.
- Engage with and respond appropriately to individuals’ questions and concerns about their MSK condition and its impact on their current situation and potentially in the future.
- Respond to individuals’ communication and information needs and support the use of accessible information where appropriate.
- Signpost individuals appropriately and effectively to sources of information and support.
- Communicate effectively with colleagues using a variety of media (e.g. verbal, written and digital) to serve individuals’ best interests.
- Respect and draw on colleagues’ knowledge and expertise within the multi-disciplinary team to serve individuals’ best interests.
- Communicate with colleagues in ways that build and sustain relationships, seeking, gathering and sharing information appropriately, efficiently and effectively to expedite and integrate individuals’ care.

## Capability 2 - Person-centred care

**Summary of comments from Round 2:**

Some of the outcomes could be reordered and there is some overlap; very important section.

**Delphi Round 2 median participant rating:** 10

**Interquartile range:** 8 to 10

**Your Delphi Round 2 rating:**

The practitioner can do the following:

- Demonstrate sensitivity to the significance of individuals’ background, culture, values and experiences for how an MSK condition impacts on their life, recognising the expertise that individuals bring to managing their own care.
- Engage with the impact of persistent pain and disability on individuals’ lives, including on their relationships, self-esteem and ability to participate in what they need and want to do (including paid and unpaid work).
- Demonstrate sensitivity to the burden of treatment for individuals with long-term MSK conditions and co-morbidities, including regular appointments that may also be for the management of their other healthcare needs.
- Progress care, recognising that reducing pain, restoring and maintaining function and independence, and improving quality of life all form clinical outcomes and meaningful goals of treatment.
- Enable individuals to make decisions about their care by helping them to identify the priorities and outcomes that are important to them.
- Enable individuals to make decisions about their care by explaining in non-technical language all available options (including doing nothing).
- Enable individuals to make decisions about their care by exploring with them the risks, benefits and consequences of each available option and discussing what these mean in the context of their life and goals.
- Enable individuals to make decisions about their care by supporting them to make a decision on their preferred way forward.

## Domain B. Assessment, Investigation and Diagnosis

MSK first point of contact practitioners conduct clinical assessments to characterise the problem and its impact and to develop differential diagnoses. This includes identifying the need for and requesting appropriate investigations and tests.

MSK first point of contact practitioners demonstrate skills in problem-solving, critical thinking and evaluating the impact and outcomes of their interventions. They analyse and synthesise information, particularly in relation to unfamiliar contexts and presentations where information may be incomplete or contradictory.

They work ethically, underpinned by their professionalism. They incorporate a critical approach to ethical uncertainty, and work actively with others to resolve ethical conflict.

MSK first point of contact practitioners demonstrate safe, effective, autonomous and reflective practice, informed by available evidence and established best practice. They work effectively as part of a team, either as a leader or as a team member, contributing to multi-disciplinary team-working to optimise the quality of service and clinical outcomes delivered to individuals. They will support and encourage shared decision e.g. working together with patients and carers to agree tests and investigations based upon clinical need and the patient’s informed preferences.

## Capability 3 – History-taking

**Summary of comments from Round 2:**

There is overlap between several of the outcomes within this capability and with aspects of other sections in the framework; some aspects (e.g. communication) dealt with elsewhere; some aspects not specific to MSK; the reference to flags is not helpful.

**Delphi Round 2 median participant rating:** 10

**Interquartile range:** 10 to 10 (no difference between data in the mid-50% of the distribution)

**Your Delphi Round 2 rating:**

The practitioner can do the following:

- Listen to individuals, ask questions and obtain appropriate additional information, with due sensitivity and consideration of what information needs to be sought to optimise the effectiveness and efficiency of the subjective examination.
- Undertake a comprehensive assessment of the issues that individuals present, taking account of how these issues relate to the presenting and past history, other determinants of health and the characteristics of MSK conditions.
- Assess the impact of individuals’ presenting symptoms, including the impairment of function, limitation of activities and restriction on participation, including work.
- Gather and synthesise information on the nature of individuals’ issues from various appropriate sources e.g. previous histories and investigations, considering how symptoms relating to the MSK system may manifest as pain, stiffness, weakness, fatigue, limitation of activities and restriction of participation.
- Explore and appraise with individuals perceptions, ideas or beliefs about their symptoms and condition and whether these may form a barrier to recovery or a return to usual activity or work.
- Appraise factors affecting individuals’ ability to participate in life situations, including work, and their perceptions of the relationship between their work and health.
- Critically appraise information obtained, taking account of the potential for MSK symptoms to be features of non-MSK conditions, indicative of serious pathology, compounded by psychological and mental health factors, and affected by lifestyle factors (including smoking, alcohol and drug misuse).
- Critically appraise complex, incomplete, ambiguous and conflicting information presented by individuals, distilling and synthesising key factors from the appraisal, and identifying those elements that may need to be pursued further.
- Record the information gathered through taking individuals’ history concisely and accurately for clinical management, and in compliance with local protocols, legal and professional requirements.

**Capability 4 - Physical assessment**

**Summary of comments from Round 2:**

GALS assessment not required; avoid listing just some of the body systems when most or all could be relevant; level of complexity needs to be revised, as some parts too advanced.

**Delphi Round 2 median participant rating:** 10

**Interquartile range:** 10 to 10 (no difference between date in the mid-50% of the distribution)

**Your Delphi Round 2 rating:**

The practitioner can do the following:

- Appropriately obtain individuals’ consent to physical examination, respect and maintain their privacy, dignity and comfort, as far as practicable, and comply with infection prevention and control procedures.
- Adapt their practice to meet the needs of different groups and individuals (including working with chaperones, where appropriate).
- Undertake observational and functional assessments of individuals relevant to their presenting condition. to identify and characterise any abnormality.
- Select and conduct an appropriate initial MSK screening assessment.
- Apply a range of physical assessment techniques appropriately, systematically and effectively, informed by an understanding of techniques’ respective validity, reliability, specificity and sensitivity and the implications of these limitations within an assessment.
- Identify, analyse and interpret potentially significant information from the physical assessment (including any ambiguities).
- Record the information gathered through assessments concisely and accurately, for clinical management and in compliance with local protocols, legal and professional requirements.

**Capability 5 - Investigations and diagnosis**

**Summary of comments from Round 2:**

Some aspects too medically orientated; including a list of conditions is problematic as there will always be some omissions; ordering investigations and tests not a reasonable expectation at this level; reconsider making recommendations for specific conditions, as other conditions could warrant similar focus.

**Delphi Round 2 median participant rating:** 10

**Interquartile range:** 10 to 10 (no difference between date in the mid-50% of the distribution)

**Your Delphi Round 2 rating:**

The practitioner can do the following:

- Assess the importance and meaning of presenting features from the clinical assessment, recognising the different patterns, syndromes and conditions commonly seen in first point of contact roles.
- Identify potential serious pathology and make appropriate onwards referral.
- Identify risk factors for severity or impact and use tools to analyse and stratify risk of progression to long-term pain and disability where available.
- Diagnose common problems that can usually be managed at first point of contact.
- Recognise and act where an early referral and diagnosis may be particularly important for optimising individuals’ long-term outcomes.
- Recognise how MSK conditions and their impact can interact with mental health, and identify when this is relevant.
- Recognise how MSK problems may be a manifestation of injury not only from trauma but also abuse, and take appropriate action when there are grounds for concern.
- Instigate appropriate investigative tests to aid diagnosis and assessment.
- Understand and interpret test results and act appropriately, demonstrating an understanding of the indications and limitations of different tests to inform decision-making and the imperative of using scarce, expensive or potentially harmful investigations judiciously.

**Domain C. Condition Management, Interventions and Prevention**

MSK first point of contact practitioners support and encourage individuals to self-manage their condition and to make behaviour changes. They focus on how they can have a positive impact on the health and wellbeing of individuals, communities and populations. They advise on interventions and therapies, and formulate and enable the development and implementation of management plans. They work in collaboration with health and social care colleagues (across services, agencies and networks) to meet individuals’ best interests.

All MSK first point of care practitioners need to be able to develop, advise on and enact an integrated management plan that considers all the options and needs and wishes of the individual, even though some of those options will be outside their scope of practice.

The MSK practitioner will support and encourage shared decision making, i.e. working together with patients and carers to select investigations, treatments, management or support packages, based upon clinical evidence of all the options and the patients informed preferences.

The management plan needs to support self-management and consider prevention, symptom control, disease control and restoration of function dependent on the possibilities and on the needs and wishes of the individual. Options to consider are education and support, lifestyle advice, drug therapies for symptom or disease control including local injections, surgery, manual techniques and rehabilitative interventions including exercise.  Capability is therefore needed by all MSK first contact practitioners in these areas.  In addition, the practitioner may have a greater level of capability related to some interventions if they are within their scope of practice.  That higher level is beyond the scope of this capability framework.

**Capability 6 - Prevention and lifestyle interventions**

**Summary of comments from Round 2:**

The health promotion aspects are desirable and should be encouraged, but should not be a pre-requisite; the word ‘understand’ cannot describe the necessary level of competence; some outcomes (i, j and k) are less important and contextual rather than required capabilities.

**Delphi Round 2 median participant rating:** 10

**Interquartile range:** 8 to 10

**Your Delphi Round 2 rating:**

The practitioner can do the following:

- Appraise the impact that a range of social, economic, and environmental factors can have on outcomes for individuals with MSK conditions, their carers and their circles of support.
- Recognise and promote the importance of social networks and communities for individuals and their carers in managing an MSK condition.
- Promote the importance of physical activity for MSK health and advise on what people with MSK conditions can and should do.
- Advise on the effects of injuries on MSK health and conditions.
- Advise on the effects of smoking, obesity and inactivity on MSK health and conditions and, where appropriate promote change or refer to relevant services.
- Advise individuals and relevant agencies on how MSK related work loss can be prevented through acting on effective risk assessments and providing appropriate working conditions, including adaptation to meet the individual’s needs.
- Use interactions to encourage changes in behaviour that can have a positive impact on the health and wellbeing of individuals, communities and populations.
- Facilitate behaviour change using evidence-based approaches that support self-management (e.g. motivational interviewing).
- Work collaboratively across agencies and boundaries to improve health outcomes and reduce health inequalities.

**Capability 7 - Self-management and behaviour change**

**Summary of comments from Round 2:**

Some overlap with other sections; having knowledge of or understand something is not sufficient, also needs to be able to do/apply; some elements do not apply equally to all levels of practitioners; some aspects more relevant for long term management of MSK conditions than to first point of contact role.

**Delphi Round 2 median participant rating:** 10

**Interquartile range:** 8 to 10

**Your Delphi Round 2 rating:**

The practitioner can do the following:

- Support individuals to self-manage and fulfil their role in their management plan, and where appropriate use principles of behaviour change theory and patient activation, to optimise their physical activity, mobility, fulfilment of personal goals and independence relevant to their MSK condition.
- Support individuals to explore the consequences of their actions and inactions on their health status and the fulfilment of their personal health goals (e.g. their engagement in exercise and their use of medication).
- Support individuals to get the most from conversations about the management of their MSK condition and its impacts by supporting and encouraging them to ask questions about what is a priority or concern for them.
- Identify risk factors for the persistence and impact of MSK conditions and help individuals manage the psycho-social implications of their condition.
- Progress their management approach with individuals recognising that MSK conditions are often coupled with mental health issues.
- Advise on and refer individuals to psychology and counselling services, in line with their needs.
- Advise individuals on the effects of their MSK condition and their response to it, including the causal links between absence from work, prolonged absence, reduced return to work and subsequent loss of employment.
- Advise and assist individuals to identify and use strategies to address work instability and to improve work retention.
- Advise on sources of relevant local or national self-help guidance, information and support.

**Capability 8 – Pharmacotherapy**

**Summary of comments from Round 2:**

The way in which some aspects are presented suggest that the practitioner is required to be a recognised prescriber; some outcomes require specialist training/skills.

**Delphi Round 2 median participant rating:** 10

**Interquartile range:** 8 to 10

**Your Delphi Round 2 rating:**

The practitioner can do the following:

- Understand the role of common medications used in managing MSK conditions, including analgesics, non-steroidal anti-inflammatory drugs, corticosteroids, drugs used in treating individuals with metabolic bone diseases, gout, inflammatory arthritis, and in the management of people with persistent pain.
- Use their understanding of the most common medications used in MSK and pain disorders to advise individuals on the medicines management of their MSK problem, the expected benefits and limitations, and inform them impartially on the advantages and disadvantages in the context of other management options.
- Identify sources of further information and advice and be able to signpost individuals as appropriate.
- Address and seek to allay individuals’ fears, beliefs and concerns.
- Keep individuals’ response to medication under review.

**Capability 9 - Injection therapy**

**Summary of comments from Round 2:**

A first point of contact practitioner does not need to be able to perform injections; for professions where injection therapy is not a core skill, the practitioner should have knowledge relating to its use and how to make appropriate referral; only a limited number of professions undertake injections routinely.

**Delphi Round 2 median participant rating:** 10

**Interquartile range:** 6-10

**Your Delphi Round 2 rating:**

The practitioner can do the following:

- Understand the role of joint injections, informed by the evidence base, in MSK practice.
- Advise on the expected benefits and limitations of injection therapy for managing an individual’s condition and inform them impartially on its advantages and disadvantages in the context of other management options.
- Address and seek to allay individuals’ fears, beliefs and concerns.
- Refer for advice about local injections, when considered appropriate.

**Capability 10 - Surgical interventions**

**Summary of comments from Round 2:**

Reviewing appropriateness for surgery should be the responsibility of the surgical team; detailed knowledge not required; reasonable to expect knowledge of procedures and when to refer on, but not to be able to fully counsel on risks and benefits.

**Delphi Round 2 median participant rating:** 10

**Interquartile range:** 7 to 10

**Your Delphi Round 2 rating:**

The practitioner can do the following:

- Understand the role of common surgical interventions used in managing MSK conditions.
- Advise on the expected benefits and limitations of most common surgical interventions used in managing specific MSK conditions where these are relevant to individuals’ care and inform them impartially on the advantages and disadvantages in the context of other management options.
- Work in partnership with individuals to explore suitability of surgical intervention, addressing and seeking to allay individuals’ fears, beliefs and concerns.
- Refer for surgical opinion when considered appropriate.

**Capability 11 - Rehabilitative interventions**

**Summary of comments from Round 2:**

Very important but very few current practitioners have appropriate training in this field; determining the benefit of these is perhaps beyond the required expertise at this level; practitioners should be comfortable delivering a home exercise programme.

**Delphi Round 2 median participant rating:** 10

**Interquartile range:** 8 to 10

**Your Delphi Round 2 rating:**

The practitioner can do the following:

- Understand the role of common rehabilitative interventions for MSK conditions.
- Advise on the expected benefits and limitations of different rehabilitative interventions used in managing specific MSK conditions, providing impartial information and advice on the advantages and disadvantages of specific interventions in the context of other management options.
- Provide basic advice on restoring function, including graded return to normal activity, navigation to self-management resources, and modifying activity for limited time periods.
- Work in partnership with individuals to explore suitability of rehabilitation interventions where appropriate.
- Prescribe personal exercise programmes to help individuals enhance, restore and maintain their mobility, function and independence.
- Refer individuals to specialist rehabilitation practitioners (e.g. occupational therapists) where this is in their best interests.
- Make recommendations to employers regarding individuals’ fitness to work, including through the appropriate use of fit notes and occupational health advice.

**Capability 12 - Interventions and care planning**

**Summary of comments from Round 2:**

Personalised exercise programmes may be too much for first contact practitioners; including a list of conditions is problematic as there will always be some omissions; practitioner should be able to avoid unnecessary onwards referrals where it will not change management or add value.

**Delphi Round 2 median participant rating:** 10

**Interquartile range:** 9 to 10

**Your Delphi Round 2 rating:**

The practitioner can do the following:

- Work in partnership with the individual to develop management plans that take account of individuals’ needs, goals and wishes, local service availability and relevant guidelines.
- Ensure the management plan considers all options that are appropriate for the care pathway.
- Advise on and instigate a management plan for common MSK conditions and their symptoms - instigating this may be through referral to others with specific relevant capabilities.
- Advise on pharmacological and non-pharmacological aspects of acute and chronic pain management.
- Advise on the links between prolonged MSK symptoms and reduced mental well-being and refer individuals to sources of mental health support when in their best interests.
- Identify when first-line intervention has been successful and discharge the patient.

**Capability 13 - Referrals and collaborative working**

**Summary of comments from Round 2:**

Key to ensuring the healthcare team is utilised appropriately; need to develop a generalist workforce, but we must not lose the unique values that each of the professions bring to their roles; very important as allows each professional role to function to its own strengths and limitations while recognising the importance of MDT collaboration to provide a comprehensive person-centred care.

**Delphi Round 2 median participant rating:** 10

**Interquartile range:** 9 to 10

**Your Delphi Round 2 rating:**

The practitioner can do the following: and Professional

- Practise within their professional and personal scope of practice and access specialist advice or support for the individual or for themselves when appropriate.
- Engage in effective inter-professional communication and collaboration with clear documentation to optimise the integrated management of the individual with an MSK condition.
- Engage in effective inter-professional communication and collaboration to optimise care for MSK conditions within the population.
- Advise on local services that individuals and their carers may benefit from accessing to help manage an MSK condition and its impact, including those relating to employment, voluntary activities, counselling services and leisure facilities.
- Draw on the expertise of all members of the multi-disciplinary team to meet individuals’ best interests and optimise the integration of their care.
- Contribute effectively to multi-disciplinary team activity (including service delivery processes and learning and development).
- Participate as an effective team member and understand the importance of effective team dynamics.
- Make appropriate referrals using appropriate documentation to other health and care professionals and agencies when this is in individuals’ best interests.

**Domain D. Service and Professional Development**

MSK first point of contact practitioners support the development of MSK services through contributing to research and evidence-based practice and evaluating the outcomes and impact of their services and interventions. They actively progress patient and public involvement in research and quality improvement activities, ensuring adherence to ethical requirements.

MSK first point of contact practitioners engage in reflective practice as part of their learning and professional development. They plan their onward personal development, taking account of self-reflection, peer review, performance feedback, and changing service needs.

**Capability 14 - Evidence-based practice and service development**

**Summary of comments from Round 2:**

These standards are fundamental to all roles in primary care, not specific to MSK; important to be aware of the evidence base; each practitioner needs to be aware of own limitations and of how to collaborate.

**Delphi Round 2 median participant rating:** 10

**Interquartile range:** 8 to 10

**Your Delphi Round 2 rating:**

The practitioner can do the following:

- Critically apply relevant national guidance and other best available evidence on MSK care and service delivery, identifying where local modifications may be required.
- Monitor and evaluate their practice and its outcomes, including through data collection and analysis to assure and improve the quality of care, service delivery and address health inequalities.
- Engage in the distinct activities of clinical audit, service evaluation and research (leading or contributing, as appropriate) adhering to the national and local requirements, and regulatory frameworks that relate to each.
- Engage in co-production initiatives with individuals and their carers to improve the person-centred design and quality of services.
- Act appropriately when services deficiencies are identified (e.g. frequent long waiting times) that have the potential to affect the effective management of individuals’ care and condition, including by taking corrective action, where needed.
- Plan, engage in and record learning and development relevant to their role and in fulfilment of professional, regulatory and employment requirements.
- Engage in reflective practice and clinical supervision as an integral part of their professional development and to inform service development and quality improvement with reference to local needs.
